# Enteric Infections, Dysbiosis, and Metabolic Dysfunction: The Role of Diarrheagenic Pathogens in Insulin Resistance

**DOI:** 10.3390/ijms27031610

**Published:** 2026-02-06

**Authors:** Martin Zermeño-Ruiz, Filiberto Gutierrez-Gutierrez, Elsa Janneth Anaya-Ambriz, Emiliano Peña-Durán, Jesús Jonathan García-Galindo, Alfredo Huerta-Huerta, Araceli Lizbeth Quiñonez-Gallardo, Daniel Osmar Suárez-Rico

**Affiliations:** 1 Doctorado en Microbiología Médica, Centro Universitario de Ciencias de la Salud (CUCS), Universidad de Guadalajara, Guadalajara 44340, Mexico; martin.zermeno@academicos.udg.mx (M.Z.-R.); elsa.anaya@academicos.udg.mx (E.J.A.-A.); 2 Departamento de Farmacobiología, Centro Universitario de Ciencias Exactas e Ingenierías, Universidad de Guadalajara, Blvd. Marcelino García Barragán #1421, Guadalajara 44430, Mexico; filiberto.gutierrez@academicos.udg.mx; 3 Departamento de Ciencias de la Salud, Centro Universitario de los Valles, Universidad de Guadalajara, Ameca 46708, Mexico; 4 Licenciatura en Médico Cirujano y Partero, Centro Universitario de Ciencias de la Salud (CUCS), Universidad de Guadalajara, Guadalajara 44340, Mexico; emilianodupe@live.com; 5 Departamento de Fisiología, Centro Universitario de Ciencias de la Salud (CUCS), Universidad de Guadalajara, Calle Sierra Mojada 950, Independencia Oriente, Guadalajara 44340, Mexico; jonathan.garcia@academicos.udg.mx; 6 Institute of Experimental and Clinical Terapeutics (INTEC), Health Science University Center, Universidad de Guadalajara, Guadalajara 44340, Mexico; 7 Hospital Medica de la Ciudad, Santa Catalina, Calle. Pablo Valdez 719, La Perla, Guadalajara 44360, Mexico; farmacovigilancia.gdl@medicadelaciudad.com (A.H.-H.); lizbeth@medicadelaciudad.com (A.L.Q.-G.)

**Keywords:** insulin resistance, diarrheagenic pathogens, gut microbiota, intestinal barrier dysfunction, short-chain fatty acids (SCFAs), chronic low-grade inflammation, *Escherichia coli*, Salmonella enterica, *Clostridioides difficile*, norovirus, rotavirus, *Giardia lamblia*

## Abstract

Type 2 diabetes and insulin resistance are increasingly recognized as conditions influenced not only by genetic and lifestyle factors but also by infectious and microbial exposures. Diarrheagenic pathogens, including enterotoxigenic, enteroaggregative, and enterohemorrhagic *Escherichia coli*, as well as other enteric microorganisms, disrupt the gut microbiota and compromise intestinal barrier integrity. These alterations promote dysbiosis, increased intestinal permeability, and systemic exposure to lipopolysaccharides and other microbial products, leading to metabolic endotoxemia and chronic low-grade inflammation. In parallel, pathogen-induced modulation of host immune responses contributes to adipose tissue inflammation, mitochondrial dysfunction, and impaired insulin signaling. This review summarizes current evidence linking diarrheagenic pathogens to insulin resistance, with emphasis on the microbiota–immune–metabolism axis. Understanding these interactions highlights novel perspectives on the pathogenesis of insulin resistance and suggests that targeted modulation of the gut microbiota or reduction in pathogen-driven inflammation may represent therapeutic opportunities to improve metabolic outcomes.

## 1. Introduction

Insulin resistance (IR) is a core metabolic abnormality that precedes and accelerates type 2 diabetes; however, beyond lifestyle and genetic determinants, it remains plausible that gut-directed infectious exposures contribute to IR by reshaping host–microbe crosstalk [1]. Diarrheagenic pathogens can perturb the gut ecosystem through convergent mechanisms that are directly relevant to insulin signaling, including sustained dysbiosis with reduced short-chain fatty acid–producing taxa and altered microbial metabolites, disruption of epithelial barrier integrity with increased permeability and translocation of microbial products (e.g., LPS), and activation of innate immune pathways that promote chronic low-grade inflammation across adipose tissue, liver, and skeletal muscle [2]. Central hypothesis: recurrent and/or severe diarrheagenic infections contribute to the initiation or worsening of IR through persistent dysbiosis, barrier dysfunction, and immunometabolic inflammation, thereby promoting metabolic endotoxemia and impaired insulin signaling. Accordingly, this narrative review synthesizes mechanistic and translational evidence linking major diarrheagenic pathogens to IR and proposes an integrative framework distinguishing shared versus pathogen-specific pathways, while highlighting key limitations that currently constrain causal inference. Given the conceptual and mechanistic scope of this article, it is presented as a narrative (non-systematic) review; therefore, PRISMA-based systematic-review procedures were not applied.

## 2. Pathophysiological Mechanisms of Insulin Resistance

Insulin resistance (IR) is a multifactorial condition characterized by a diminished ability of peripheral tissues, primarily skeletal muscle, liver, and adipose tissue, to respond adequately to the action of insulin [1]. This alteration results from a complex interplay of metabolic, inflammatory, and cellular stress related mechanisms that impair intracellular insulin signaling [1]. Under physiological conditions, insulin regulates energy metabolism by promoting glucose uptake in muscle and adipose tissue, stimulating glycogen and lipid synthesis, and inhibiting both hepatic gluconeogenesis and lipolysis [3].

**Figure 1:**
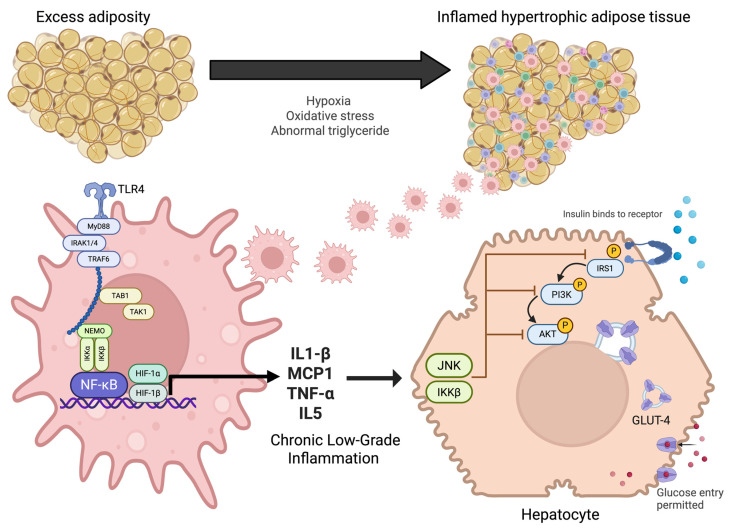
Pathophysiological mechanisms linking adipose tissue inflammation to insulin resistance. Excess adiposity promotes adipocyte hypertrophy, hypoxia, oxidative stress, and abnormal triglyceride accumulation, leading to inflamed adipose tissue. This activates innate immune signaling through TLR4 and NF-κB pathways in macrophages, resulting in the release of pro-inflammatory cytokines (IL-1β, MCP1, TNF-α, IL-5). Chronic low-grade inflammation interferes with insulin receptor signaling in hepatocytes via JNK and IKKβ, impairing IRS1/PI3K/AKT activation. This disruption reduces GLUT-4 translocation and limits glucose uptake, thereby contributing to systemic insulin resistance.

However, in states of insulin resistance, these processes are disrupted: glucose uptake is impaired, hepatic glucose production is not adequately suppressed, and lipolysis is intensified, all of which contribute to persistent hyperglycemia [4]. At the molecular level, insulin signaling begins with its binding to the insulin receptor, triggering a cascade of events that include receptor autophosphorylation and the activation of adaptor proteins such as IRS, PI3K, and Akt. This signaling pathway is essential for the translocation of glucose transporters (GLUT4) to the plasma membrane and for the regulation of key enzymes involved in glucose and lipid metabolism (Figure 1) [3].

Moreover, in IR states, abnormal serine phosphorylation of IRS proteins has been reported, which disrupts downstream signaling and contributes to metabolic dysfunction. This impairment leads to compensatory hyperinsulinemia which, when sustained over time, further aggravates the metabolic imbalance. Thus, insulin resistance not only precedes the development of type 2 diabetes (T2D) but also plays a central role in its pathophysiology [5].

## 3. Alterations in Insulin Signaling Due to Ectopic Lipid Accumulation

Ectopic lipids refer to fat deposits that accumulate in organs not specialized for lipid storage, such as the liver, skeletal muscle, heart, and pancreas. This accumulation is often a consequence of chronic nutrient excess or dysfunction of adipose tissue [6]. Under normal conditions, adipocytes uptake fatty acids and convert them into triglycerides, which are stored as lipid droplets. However, when the storage capacity of adipose tissue is exceeded either due to persistent overnutrition or disorders such as lipodystrophy there is an increased release of fatty acids into the bloodstream. These circulating lipids are then taken up by other tissues, where they accumulate in a non-physiological manner [7]. Although initially this process may represent an adaptive response, prolonged accumulation promotes the generation of lipid intermediates that disrupt key cellular functions, including insulin signaling [6].

IR is closely linked to abnormal triglyceride (TAG) accumulation in peripheral tissues, where TAG synthesis plays a key role. This process begins with fatty acid activation by acyl-CoA synthetases and proceeds through key enzymes in the glycerol-3-phosphate pathway, including glycerol-3-phosphate acyltransferase (GPAT), acylglycerol-3-phosphate acyltransferase (AGPAT), lipins (phosphatidic acid phosphatases), and diacylglycerol acyltransferase (DGAT). While TAGs serve as neutral lipids stored in lipid droplets and do not directly regulate insulin signaling, lipid intermediates generated in this pathway, such as diacylglycerol (DAG) and phosphatidic acid (PA), negatively modulate insulin signaling [8].

Specifically, phosphatidic acid (PA) derived from GPAT activity impairs the mTORC2 complex, inhibiting Akt activation, while diacylglycerol (DAG) activates specific protein kinase C (PKC) isoforms that interfere with insulin receptor substrate-1 (IRS1) function [9]. Other metabolites such as ceramides, acyl-CoA, and lysophosphatidic acid (LPA) also disrupt insulin signaling by impairing glucose uptake and the phosphorylation of key proteins involved in maintaining metabolic homeostasis [10]. Therefore, enzymatic regulation of the glycerol-3-phosphate pathway is critical to understanding the molecular mechanisms underlying insulin resistance and offers potential targets for therapeutic intervention in metabolic diseases.

## 4. Inflammatory and Stress-Activated Pathways Disrupting Insulin Signaling: The IRS–PI3K–Akt, JNK, and IKKβ

Beyond the accumulation of lipid intermediates, there is increasing evidence supporting the role of chronic low-grade inflammation and cellular stress responses as key disruptors of insulin signaling. Pro-inflammatory cytokines such as tumor necrosis factor-alpha (TNF-α), interleukin-6 (IL-6), and interleukin-1β (IL-1β), which are elevated in insulin-resistant (IR) states, activate intracellular stress-associated kinases, particularly JNK (c-Jun N-terminal kinase) and IKKβ (IκB kinase β) [11]. These kinases promote the serine phosphorylation of insulin receptor substrates (IRSs), impairing their ability to interact with the insulin receptor and with PI3K. This modification not only attenuates downstream signaling but also facilitates proteasomal degradation of IRS proteins, further decreasing insulin sensitivity [12].

IKKβ activation additionally triggers the NF-κB signaling pathway, amplifying the transcription of pro-inflammatory genes and establishing a self-perpetuating inflammatory cycle within insulin-sensitive tissues. In parallel, JNK activation is often associated with oxidative stress and mitochondrial dysfunction exacerbates insulin resistance by interfering with Akt phosphorylation. These stress pathways are particularly prominent in adipose tissue, where hypertrophic adipocytes and infiltrating macrophages generate a pro-inflammatory microenvironment [13]. Recent studies have also identified endoplasmic reticulum (ER) stress as a relevant contributor, particularly through the unfolded protein response (UPR), which intersects with the JNK and IKKβ pathways, further aggravating the impairment of insulin signaling [14].

Mitochondrial dysfunction and oxidative stress (O_2_^•−^, H_2_O_2_) are directly implicated in IR by specifically inhibiting GLUT4 transport without necessarily affecting mitochondrial respiration [15]. LPS-induced inflammation transiently disrupts mitochondrial biogenesis and mitophagy in muscle, affecting regulatory factors such as BNIP3 and BNIP3L, although without significantly altering overall mitochondrial content. Excessive mitochondrial fission mediated by Drp1 contributes to muscle IR through ROS production, lipotoxicity, and mTOR-S6K pathway activation inhibiting IRS1. Advanced techniques like 31P-MRS and BOLD MRI reveal that mitochondrial oxidative capacity in skeletal muscle remains unaffected in insulin resistance, though significant deficits in muscle oxygenation are present. These factors—oxidative stress, altered mitophagy, excessive mitochondrial dynamics, and tissue oxygenation deficits—are closely connected with muscular IR in inflammatory and metabolic contexts [15,16].

Furthermore, the gut microbiota has been recognized as a critical regulator of metabolic inflammation and insulin sensitivity. Dysbiosis, or microbial imbalance, promotes intestinal barrier dysfunction and facilitates systemic translocation of lipopolysaccharides (LPSs), which are potent activators of Toll-like receptor 4 (TLR4) [17]. TLR4 activation in metabolic tissues stimulates IKKβ and JNK signaling, contributing to impaired insulin action [18]. Experimental models have demonstrated that both acute and chronic exposure to LPSs is sufficient to induce insulin resistance through these pathways. Collectively, inflammation, cellular stress, and microbiome-derived signals converge on common molecular nodes IRS, PI3K, Akt, JNK, and IKKβ to disrupt insulin signaling and perpetuate metabolic dysfunction [19].

## 5. Endoplasmic Reticulum Stress and Insulin Resistance

The ER regulates protein folding, calcium homeostasis, and lipid synthesis. Under conditions of nutrient overload, hyperglycemia, or excess saturated fatty acids, misfolded proteins accumulate, triggering an adaptive response known as ER stress or the unfolded protein response (UPR). Sustained UPR activation induces signaling pathways such as IRE1–JNK and PERK–eIF2α, which interfere with insulin signaling. In insulin-sensitive tissues such as the liver, skeletal muscle, and adipose tissue, this leads to IRS-1 serine phosphorylation and impaired insulin sensitivity [20].

ER stress is exacerbated by inflammatory and microbial signals, including lipopolysaccharides (LPSs) derived from intestinal dysbiosis or enteric infections [21]. These signals promote metabolic endotoxemia, epithelial dysfunction, and both local and systemic inflammation. Moreover, UPR activation in immune cells, such as infiltrating macrophages, enhances the secretion of pro-inflammatory cytokines. This immunometabolic axis sustains chronic low-grade inflammation and impairs insulin signaling. Thus, the ER constitutes a key regulatory node in the pathophysiology of insulin resistance [22].

## 6. Chronic Low-Grade Inflammation and Insulin Resistance

Chronic low-grade inflammation is a persistent and silent state characteristic of multiple metabolic diseases, including insulin resistance. It partly originates from adipose tissue dysfunction, where hypertrophied adipocytes secrete pro-inflammatory cytokines such as TNF-α, IL-6, and IL-1β. These molecules disrupt insulin signaling by activating pathways such as JNK, IKKβ, and NF-κB. Additionally, sustained inflammation promotes oxidative stress and recruitment of M1 macrophages, amplifying metabolic homeostasis disturbances [23].

Recent studies have demonstrated that low-grade inflammation is also linked to epigenetic alterations and cellular senescence, exacerbating insulin resistance. Factors such as increased interleukin-1 and activation of the NLRP3 inflammasome have been implicated in inhibiting the IRS–PI3K–Akt axis. Furthermore, peripheral tissues like skeletal muscle and liver show immune infiltration and mitochondrial dysfunction. This systemic inflammatory environment constitutes a key mechanism in the progression to T2D [24].

## 7. Diarrheagenic Pathogens and Their Impact on Metabolism: Relationship with Insulin Resistance

**Figure 2:**
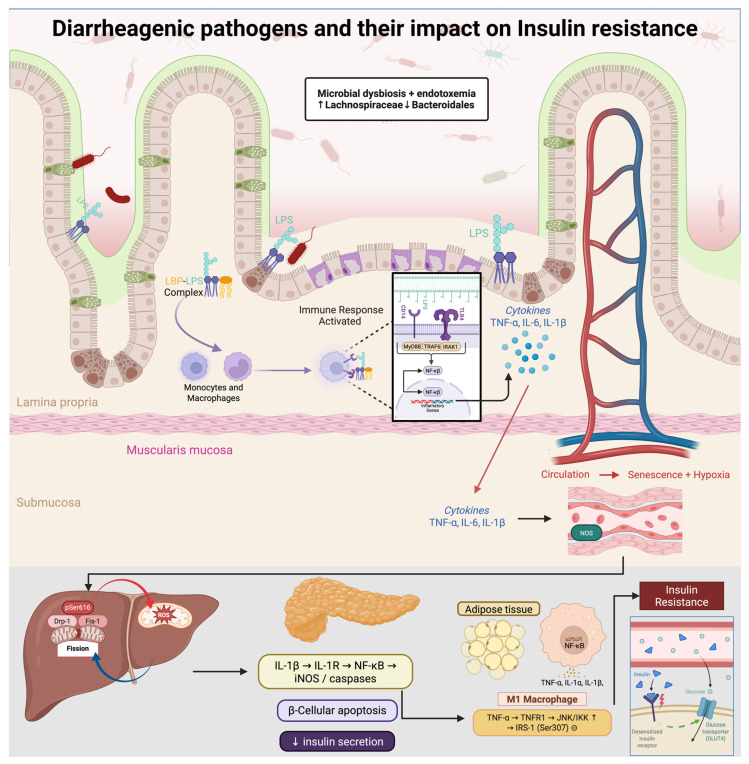
Diarrheagenic pathogens drive gut dysbiosis (↑ Lachnospiraceae, ↓ Bacteroidales) and metabolic endotoxemia. Circulating LPS, complexed with LBP, activates CD14/TLR4–MyD88/IRAK/TRAF6 signaling, inducing NF-κB and secretion of TNF-α, IL-6, and IL-1β. These cytokines promote M1 polarization and inhibit insulin signaling via JNK/IKK–mediated inhibitory phosphorylation of IRS-1 (Ser307). In skeletal muscle, Drp1-pSer616–driven mitochondrial fission elevates ROS and activates mTOR–S6K, further suppressing IRS-1; chronic inflammation also causes endothelial senescence and hypoxia. In pancreatic β-cells, IL-1β triggers iNOS/caspases and apoptosis, reducing insulin secretion. Net effect: decreased Akt activation, impaired GLUT4 translocation, and systemic insulin resistance.

The human gastrointestinal tract constitutes a complex ecosystem where diverse commensal microorganisms coexist with the intestinal epithelium, associated immune tissue, and a dense network of neuroendocrine signaling pathways [25]. The homeostasis of this system is crucial for maintaining digestive, immune, and metabolic functions [26]. However, infection by enteric pathogens can destabilize this balance, triggering both local and systemic effects (Figure 2) [25].

In this context, gastrointestinal infections caused by bacteria, viruses, and parasites not only represent a major global health burden due to direct microbial infection, but are also associated with other pathologies such as cancer, inflammatory bowel disease, food hypersensitivity, autoimmune disorders, reactive arthritis, cholecystitis, pancreatitis, colitis, and type 2 diabetes [27].

Although there are multiple factors involved in the development of these pathologies, it has been shown that infections by various diarrheagenic pathogens such as *Escherichia coli*, *Salmonella enterica*, *Clostridioides difficile*, Norovirus, Rotavirus and *Giardia lamblia* can induce alterations in the gut-metabolism axis [28].

Across diarrheagenic infections, three recurring processes plausibly link enteric disease to insulin resistance (IR): (i) dysbiosis with depletion of short-chain fatty acid (SCFA)–producing taxa and reduced butyrate/propionate availability, (ii) intestinal barrier dysfunction with increased permeability and translocation of microbial products (e.g., LPSs), and (iii) cytokine-driven immunometabolic inflammation (e.g., TNF-α, IL-6) that interferes with insulin signaling pathways (see Section 4 and Section 6). In the pathogen-specific sections below, we emphasize organism-specific features and avoid repeating this shared framework [29].

### 7.1. Escherichia coli

**Figure 3:**
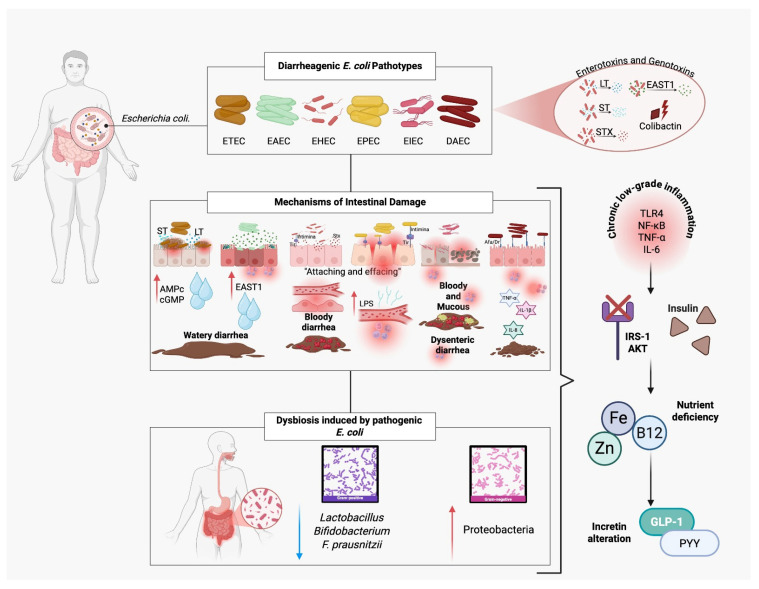
Pathogenic mechanisms of diarrheagenic *Escherichia coli* and their contribution to insulin resistance. Diarrheagenic *E. coli* pathotypes (ETEC, EAEC, EHEC, EPEC, EIEC, DAEC) produce enterotoxins (LT, ST, EAST1) and genotoxins (colibactin) that disrupt intestinal function, causing watery, bloody, or dysenteric diarrhea. EPEC and EHEC induce “attaching and effacing” lesions, damaging microvilli and altering tight junctions, which facilitate lipopolysaccharide (LPS) translocation. Pathogenic *E. coli* also triggers dysbiosis, reducing beneficial bacteria (*Lactobacillus*, *Bifidobacterium*, *F. prausnitzii*) and increasing Proteobacteria. These alterations lead to chronic low-grade inflammation (↑ TLR4, NF-κB, TNF-α, IL-6), impaired insulin signaling (IRS-1/AKT pathway), nutrient deficiencies (zinc, iron, vitamin B12), and reduced incretin secretion (GLP-1, PYY), ultimately promoting insulin resistance.

*Escherichia coli* (*E. coli*) is a Gram-negative, facultatively anaerobic, rod-shaped bacterium that primarily resides in the intestinal tract and is considered part of the normal gut microbiota. However, certain pathotypes possess virulence determinants that confer pathogenic potential, making *E. coli* responsible for a significant proportion of diarrheal diseases. Among these, the ETEC (enterotoxigenic), EAEC (enteroaggregative), EHEC (enterohemorrhagic, Shiga toxin-producing), EPEC (enteropathogenic), EIEC (enteroinvasive), and DAEC (diffusely adherent) pathotypes stand out for their pathogenicity, particularly in children and in areas with poor sanitation infrastructure (Figure 3) [27].

In addition to diarrheal illness, some *E. coli* strains can produce colibactin, a toxin that has been shown to alkylate DNA, causing double-strand breaks and specific mutations that may contribute to colorectal carcinogenesis. These strains are therefore considered genotoxic [30].

#### 7.1.1. Alteration of the Gut Microbiota

Infection with diarrheagenic *E. coli* leads to profound disruption of the intestinal ecosystem. Upon colonizing the small or large intestine, these bacteria displace commensal species through niche competition, binding to common receptors, and secreting antimicrobial compounds such as microcins and siderophores. Additionally, certain pathotypes release toxins such as EAST1 and LT/ST, which alter the intestinal microenvironment—pH, water content, and ion balance—making it inhospitable for beneficial bacteria [31].

Metagenomic studies have shown that pathogenic *E. coli* infections significantly reduce the abundance of anti-inflammatory, short-chain fatty acid producing genera such as *Lactobacillus*, *Bifidobacterium*, and *Faecalibacterium prausnitzii*. This dysbiosis is often accompanied by an increase in Proteobacteria, a hallmark of proinflammatory intestinal states [32].

The reduction in short-chain fatty acids such as butyrate and propionate affects multiple metabolic functions, including the inhibition of G-protein-coupled receptors (GPR41, GPR43), which are involved in incretin (GLP-1, PYY) secretion. This disrupts the gut–pancreas axis, impairing postprandial insulin release and contributing to altered lipid metabolism and reduced insulin sensitivity in hepatic and muscular tissues [33].

Furthermore, *E. coli*-induced dysbiosis may impair the bacterial synthesis of B vitamins, including folate (B9) and cobalamin (B12), which are essential for mitochondrial function, epigenetic regulation, and energy homeostasis. Their deficiency is associated with insulin resistance and disruption of AMPK signaling in peripheral tissues [34].

#### 7.1.2. Intestinal Barrier Damage

Certain diarrheagenic *E. coli* strains can directly damage the intestinal epithelial architecture. EPEC, for instance, adheres tightly to the epithelium via the intimin protein, which interacts with the translocated receptor Tir. This interaction triggers cytoskeletal rearrangement in enterocytes, forming “attaching and effacing” lesions in which microvilli are literally erased. This reduces the absorptive surface and compromises epithelial mechanical integrity. It also facilitates the translocation of bacterial products such as lipopolysaccharide (LPS) into the bloodstream, potentially inducing low-grade systemic inflammation—a key contributor to insulin resistance [35].

Additionally, EHEC and EPEC reduce the expression of tight junction proteins such as claudin-1, occludin, and ZO-1. This functional loss of intercellular junctions results in increased intestinal permeability (commonly referred to as “leaky gut”), allowing paracellular passage of immunogenic molecules such as LPS, flagellin, and peptidoglycans [35].

This microbial translocation activates Toll-like receptors (TLR4, TLR5) in the submucosal tissue, triggering NF-κB signaling and systemic production of cytokines such as TNF-α and IL-6. These cytokines promote insulin resistance by interfering with IRS-1 and AKT signaling in adipose, hepatic, and muscle tissues. Increased permeability is also associated with upregulation of zonulin, a tight junction regulator whose overexpression has been linked to chronic metabolic diseases and autoimmunity [36].

#### 7.1.3. Impaired Nutrient Absorption

The integrity of the intestinal epithelium is essential for the proper absorption of nutrients. Therefore, epithelial damage, microvilli atrophy, local inflammation, and alteration of intestinal transporters lead to defective absorption of several essential micronutrients, significantly reducing the uptake of key elements such as zinc and iron [37].

Zinc: Zinc is crucial for the function of antioxidant enzymes such as superoxide dismutase (SOD), for insulin receptor signaling, and for the expression of the transcription factor PPAR-γ. Under conditions of dysbiosis and inflammation, decreased expression of the ZnT8 transporter (Slc30A8) has been reported, reducing zinc uptake at both intestinal and pancreatic levels. Zinc deficiency is directly associated with impaired insulin secretion and increased cellular oxidative stress [38].

Iron: Chronic intestinal inflammation decreases the expression of the DMT1 transporter and ferroportin, limiting intestinal iron absorption. This may lead to functional anemia, impairing tissue oxygenation and energy metabolism in insulin-sensitive tissues [39].

### 7.2. Salmonella enterica

*Salmonella enterica* is a Gram-negative, facultatively anaerobic bacterium belonging to the Enterobacteriaceae family. It is one of the leading causes of gastroenteritis worldwide. The species is divided into more than 2600 serotypes, with *S.* Typhimurium and *S.* Enteritidis being the most associated with non-typhoidal enteric diseases in humans [40].

Transmission typically occurs through consumption of contaminated food, particularly poultry products, raw eggs, and improperly washed vegetables. These infections pose a significant burden in terms of morbidity and mortality; *Salmonella* is estimated to cause over 93 million infections and 155,000 deaths annually, especially in developing countries and among vulnerable populations such as young children, the elderly, and immunocompromised individuals [41].

Following the ingestion of contaminated food or water, *Salmonella* colonizes the small intestine—particularly the terminal ileum—where it triggers a series of immunological and pathophysiological responses that affect not only local epithelial integrity but also the host’s systemic metabolic function [42].

The main mechanism of *Salmonella* invasion involves type III secretion systems (SPI-1 and SPI-2), which enable the injection of effector proteins into intestinal epithelial cells. These proteins reorganize the host cytoskeleton, facilitating the active invasion of enterocytes and M cells in the ileal epithelium. The bacterium also induces apoptosis, triggers the release of inflammatory cytokines (IL-8, TNF-α, IL-1β), and activates inflammatory pathways mediated by TLRs and NLRs, contributing to a persistent proinflammatory environment [43].

#### 7.2.1. Alteration of the Gut Microbiota

One of the most significant effects of *Salmonella enterica* infection is the disruption of the intestinal microbiota balance. This bacterium has developed strategies to exploit the inflammatory environment it induces, displacing commensal bacteria and promoting the expansion of other pro-inflammatory species [44].

In both murine models and human studies, *S.* Typhimurium infection has been shown to rapidly reduce microbial diversity, along with a decline in the abundance of *Clostridium*, *Faecalibacterium*, and Bacteroidetes—groups associated with anti-inflammatory effects and the promotion of immune tolerance. At the same time, it increases the relative proportion of Proteobacteria, thereby exacerbating the pro-inflammatory state induced by *Salmonella*, a hallmark pattern of intestinal dysbiosis [45].

This shift is partly mediated by neutrophilic inflammation, which leads to the release of reactive oxygen (ROS) and nitrogen species (RNS). These molecules create an oxidative environment in the intestinal lumen that suppresses obligate anaerobes while enabling the expansion of facultative bacteria like *Salmonella* and other Enterobacteriaceae [46,47].

Consistent with the shared dysbiosis–barrier disruption–inflammation axis described above, this loss of SCFA-producing taxa and expansion of Proteobacteria provides a plausible route to impaired insulin sensitivity.

#### 7.2.2. Intestinal Barrier Damage

*Salmonella* possesses a type III secretion system (TTSS), encoded within pathogenic islands SPI-1 and SPI-2, which enables the injection of effector proteins into intestinal epithelial cells. These proteins reorganize the actin cytoskeleton, facilitating bacterial internalization. This process is associated with multiple forms of epithelial damage, including disruption of intercellular junctions, increased paracellular permeability, bacterial translocation, and abnormal epithelial apoptosis and regeneration [48,49,50].

Intercellular junction disruption: Infection leads to decreased expression and altered distribution of proteins (e.g., occludin, claudin-1, and ZO-1) resulting in a loss of barrier function and unregulated passage of macromolecules, antigens, and microbial products from the intestinal lumen into the subepithelial space [51,52].

Apoptosis and abnormal epithelial renewal: *Salmonella* effector proteins also induce apoptosis in enterocytes and M cells, further compromising epithelial integrity and reducing regenerative capacity [53,54].

Together, these effects not only predispose the host to chronic intestinal inflammation but also promote conditions that impair insulin signaling via immunometabolic mechanisms.

#### 7.2.3. Impaired Nutrient Absorption

The structural and functional damage to the intestinal epithelium caused by *Salmonella* infection has direct consequences on the intestine’s ability to absorb essential nutrients involved in energy metabolism and insulin regulation [55].

- Zinc: *Salmonella* reduces the expression of the ZIP4 transporter (SLC39A4), which mediates zinc uptake in the small intestine. This leads to decreased insulin synthesis and secretion by pancreatic β-cells and weakens cellular antioxidant defenses. Zinc deficiency promotes oxidative stress and mitochondrial dysfunction—two processes associated with insulin resistance [56,57].
- Iron: *Salmonella* disrupts iron homeostasis, inhibition of the DMT1 transporter (Divalent Metal Transporter 1) and upregulation of hepcidin, the regulatory hormone that blocks iron export. Iron is vital for mitochondrial function and cellular energy efficiency. Its functional deficiency can lead to metabolic fatigue, impaired mitochondrial biogenesis, and insulin resistance [58].

### 7.3. Clostridium difficile

*Clostridium difficile* is a Gram-positive, spore-forming, strictly anaerobic bacterium belonging to the class Clostridia. It is considered one of the primary etiological agents of antibiotic-associated diarrhea and pseudomembranous colitis. This opportunistic pathogen colonizes the intestine following disruption of the microbiota by antimicrobials, particularly in hospital settings and in patients with comorbidities. *C. difficile* is recognized as the most common cause of diarrhea associated with broad-spectrum antibiotics, which eliminate commensal microbiota and allow for the overgrowth of toxigenic strains [59,60].

The infection can range from mild diarrhea to potentially fatal pseudomembranous colitis. In developed countries, its impact has increased, exacerbated by hypervirulent strains (e.g., ribotype 027/NAP1/BI). *C. difficile* produces two major exotoxins: TcdA and TcdB. Both act by glucosylating cytoskeletal GTPases (Rho, Rac, Cdc42), causing cytoskeletal disorganization, loss of cell junctions, apoptosis, and massive inflammatory responses. These toxins also induce fluid secretion and pro-inflammatory cytokines, contributing to diarrhea and tissue damage [61,62,63].

#### 7.3.1. Gut Microbiota Disruption

As previously discussed, dysbiosis is a central event in the pathophysiology of many infections, including *C. difficile*. This bacterium rarely causes disease in individuals with a diverse and stable gut microbiota. However, after exposure to broad-spectrum antibiotics (such as clindamycin, cephalosporins, fluoroquinolones), a significant loss of commensal bacteria occurs, creating an ecological niche for spore germination and bacterial proliferation [64,65].

Microbiome sequencing studies in patients with *C. difficile* infection have shown a notable reduction in the families Lachnospiraceae, Ruminococcaceae, and Bacteroidaceae—key short-chain fatty acid producers, particularly butyrate. In parallel, there is an increase in pathogenic taxa such as *Enterococcus*, *Escherichia*, and other *Proteobacteria*, which exacerbate inflammation and the production of toxic metabolites [65,66]. Consistent with the shared dysbiosis–barrier disruption–inflammation axis described above, this dysbiotic pattern provides a plausible route to impaired metabolic regulation.

#### 7.3.2. Intestinal Barrier Damage

The intestinal epithelium forms a physical and functional barrier separating the luminal content from the internal milieu. In *C. difficile* infection, this barrier is severely compromised, largely due to the direct action of TcdA and TcdB. These toxins are glucosyltransferases that modify cytoskeletal GTPases (Rho, Rac, Cdc42), disrupting intercellular junctions, inducing apoptosis, and causing epithelial sloughing.

Structural damage includes the disruption of tight junctions, via reduced expression of key proteins such as claudin-1, occludin, and ZO-1, resulting in increased paracellular permeability, inflammation, and mucosal necrosis. This leads to the release of proinflammatory cytokines (IL-1β, TNF-α, IL-8), which perpetuate epithelial damage, promote neutrophil migration, and contribute to pseudomembrane formation. Additionally, epithelial regeneration is impaired through inhibition of the Wnt/β-catenin pathway—critical for the proliferation of intestinal stem cells—delaying mucosal repair [67,68].

The net result is a highly permeable intestinal barrier, allowing the translocation of bacterial antigens such as LPS, peptidoglycan fragments, and lipoteichoic acids into the systemic circulation. This induces chronic activation of the innate immune system via NF-κB signaling and elevates IL-6 and TNF-α levels—mediators directly involved in insulin resistance through inhibition of the IRS-1/PI3K/AKT pathway. This low-grade persistent inflammatory state is also linked to increased risk of metabolic syndrome, non-alcoholic fatty liver disease, and microbial dysbiosis in peripheral tissues such as adipose tissue [69,70,71]. Similarly to other infections, *C. difficile* impairs the absorption of zinc, which is essential for insulin secretion, antioxidant defense, and mitochondrial function [72,73].

### 7.4. Norovirus

Norovirus is a non-enveloped, positive-sense, single-stranded RNA virus belonging to the Caliciviridae family. It is the leading cause of acute viral gastroenteritis in adults worldwide. Its epidemiological relevance has increased in recent decades due to its low infectious dose (<20 viral particles), high environmental resistance, and ability to cause massive outbreaks in closed communities such as schools, hospitals, cruise ships, and nursing homes. Transmission occurs mainly via the fecal-oral route, direct contact, or contaminated fomites, and the virus can survive on surfaces for several days [74,75].

Norovirus infects and destroys mature enterocytes in the small intestine, leading to villous atrophy and a reduction in absorptive surface area. This results in a malabsorption syndrome and secretory diarrhea, sometimes accompanied by severe vomiting. It also affects intestinal motility and the secretion of digestive enzymes. Although considered a self-limiting pathogen, recent evidence shows that alterations in microbiota composition, intestinal epithelial integrity, and systemic metabolism can persist even after clinical recovery [76,77].

#### 7.4.1. Alteration of the Gut Microbiota

Norovirus infection can induce changes in the intestinal microbiota, particularly in individuals with repeated infections, those who are immuno-compromised, or those with preexisting microbiota disturbances. Although typically milder and more transient than bacterial infections, the microbial shifts can still be clinically significant [78,79].

Metagenomic studies have identified a decrease in microbial richness and alpha diversity following norovirus infection, with reductions in beneficial genera such as *Faecalibacterium prausnitzii*, *Bifidobacterium*, *Lactobacillus*, *Roseburia*, and *Akkermansia muciniphila*—all known for their anti-inflammatory properties and production of short-chain fatty acids. Conversely, there is an increase in opportunistic bacteria such as Enterobacteriaceae, Streptococcus, and Escherichia [78,80,81].

#### 7.4.2. Intestinal Barrier Damage

Norovirus infection directly affects the epithelial architecture of the small intestine by targeting mature enterocytes located on the villi, without invading the basal epithelium. This cellular destruction impairs absorptive function and induces barrier disruption that may persist beyond the acute phase [82,83].

Villous shortening: The loss of functional enterocytes reduces the absorptive surface, negatively impacting nutrient digestion and absorption [84].

Tight junction disruption: The infection reduces the expression of proteins such as claudin-1, occludin, and zonulin, compromising epithelial cohesion and allowing the non-selective passage of luminal macromolecules into the submucosa [85].

Together, these events increase intestinal permeability and facilitate the systemic dissemination of microbial products, contributing to low grade inflammation that may impair insulin signaling [86,87].

In individuals with genetic predispositions or pre-existing conditions (e.g., obesity, metabolic syndrome), this proinflammatory state may persist and act as an amplifier of insulin resistance [88].

#### 7.4.3. Impaired Nutrient Absorption

The structural damage to the intestinal epithelium induced by norovirus has significant functional consequences for the absorption of nutrients essential to energy metabolism, including zinc, crucial for immune function and insulin sensitivity, and B-complex vitamins (B1, B6, B9, B12), necessary for mitochondrial energy metabolism and epigenetic regulation [40,89].

These changes directly affect immunometabolic homeostasis, promoting chronic inflammation, an increase in pro-inflammatory bacterial species, and nutrient absorption deficiencies [90,91]. This is especially critical in individuals with persistent post-norovirus dysbiosis, where sustained reductions in beneficial microbial metabolites and elevations in pro-inflammatory metabolites drive chronic intestinal immunometabolic dysregulation [29,92].

### 7.5. Rotavirus

Rotavirus is a segmented, double-stranded RNA virus which is non-enveloped, belonging to the genus Rotavirus within the Reoviridae family. It is recognized as the most common cause of severe viral gastroenteritis in infants and young children worldwide. Before the widespread introduction of the vaccine, it was estimated to cause around 500,000 child deaths annually. Although its impact has been drastically reduced in regions with high vaccination coverage, it remains a significant cause of morbidity in low- and middle-income countries. Transmission is fecal-oral and highly contagious [93,94,95].

Rotavirus infects and destroys mature enterocytes located at the tips of the villi in the small intestine, impairing absorption and triggering both osmotic and secretory diarrhea. The viral protein NSP4, which acts as an enterotoxin, disrupts intracellular calcium levels, altering the secretion of electrolytes and water. It also contributes to the activation of pro-inflammatory mechanisms that damage epithelial architecture [96,97,98].

#### 7.5.1. Alteration of Gut Microbiota

During acute rotavirus infection, there is a marked disturbance in the gut microbiota, especially in pediatric populations whose microbial communities are still developing. This dysbiosis may have short- and long-term effects, depending on the severity of the infection, the host’s nutritional status, and the recurrence of the virus [99,100,101].

A reduction in the diversity and abundance of *Faecalibacterium*, *Lactobacillus*, and *Bifidobacterium* has been observed. These bacteria play roles in modulating intestinal inflammation, immune function, and energy homeostasis. Their decline results in impaired epithelial repair and increased vulnerability to metabolic endotoxemia, a condition in which bacterial products cross the intestinal barrier and trigger systemic inflammatory responses. Simultaneously, an increase in *Proteobacteria*, particularly *Escherichia*/*Shigella*, has been noted, favoring inflammation and leading to a reduction in short-chain fatty acids, particularly butyrate [101,102,103,104].

Collectively, these post-rotavirus microbiota changes impair the gut-metabolism axis and may predispose individuals to long-term conditions such as insulin resistance, particularly in those exposed early in life and consuming low-fiber or micronutrient-deficient diets [103,105].

#### 7.5.2. Damage to the Intestinal Barrier

Rotavirus primarily infects mature enterocytes of the small intestinal villi, leading to their lysis and detachment. This destruction reduces villus height and results in incomplete mucosal regeneration, particularly in cases of reinfection or malnutrition. It causes disruption of intercellular junctions, where the viral protein NSP4, with enterotoxic activity, alters intracellular calcium transport, compromising the structure of tight junctions by reducing the expression of key proteins such as claudin-1, occludin, and zonulin. This facilitates the passage of antigens and toxins into the subepithelial space [96,97,98]. Together, these epithelial alterations can increase permeability and favor low-grade inflammatory responses that may impair insulin sensitivity, particularly in susceptible hosts [103,105,106].

#### 7.5.3. Reduced Nutrient Absorption

Rotavirus-induced structural damage directly impairs the intestine’s ability to absorb and transport nutrients, including glucose [107,108], zinc [109,110], and vitamins A, B, D, E, and K [111,112], as well as the production and absorption of short-chain fatty acids [113,114]. This has profound implications for energy and endocrine metabolism [96,115].

For instance, post-infection zinc deficiency has been documented in children with rotavirus and has been linked to growth impairments and altered insulin responses [109,110], while rotavirus infections reduce the intestine’s ability to absorb vitamins such as B9 and B12, especially in cases of chronic villus damage [96].

### 7.6. Giardia lamblia

*Giardia lamblia* (also known as *G. intestinalis* or *G. duodenalis*) is an anaerobic flagellated protozoan that colonizes the proximal portion of the small intestine. It exists in two forms: a cyst (infective) and a trophozoite (active). It is one of the most common intestinal pathogens worldwide, particularly in areas with limited access to clean water, basic sanitation, and among vulnerable populations such as children, travelers, and immunocompromised individuals. It is estimated to affect over 200 million people annually, with the highest prevalence among children, travelers, and rural populations. The infection, known as giardiasis, can be acute or chronic, and although it is often self-limiting, it has the potential to induce persistent alterations in intestinal physiology and the gut-metabolism axis [28,116,117].

*Giardia adheres* to the epithelial surface of the small intestine via a ventral adhesive disc, without invading the tissue. Its presence triggers epithelial dysfunction, enterocyte apoptosis, altered intestinal motility, and a moderate inflammatory response, especially in prolonged infections. The secretion of proteases and other virulence factors also contributes to tissue damage [118,119].

#### 7.6.1. Alteration of the Gut Microbiota

**Figure 4:**
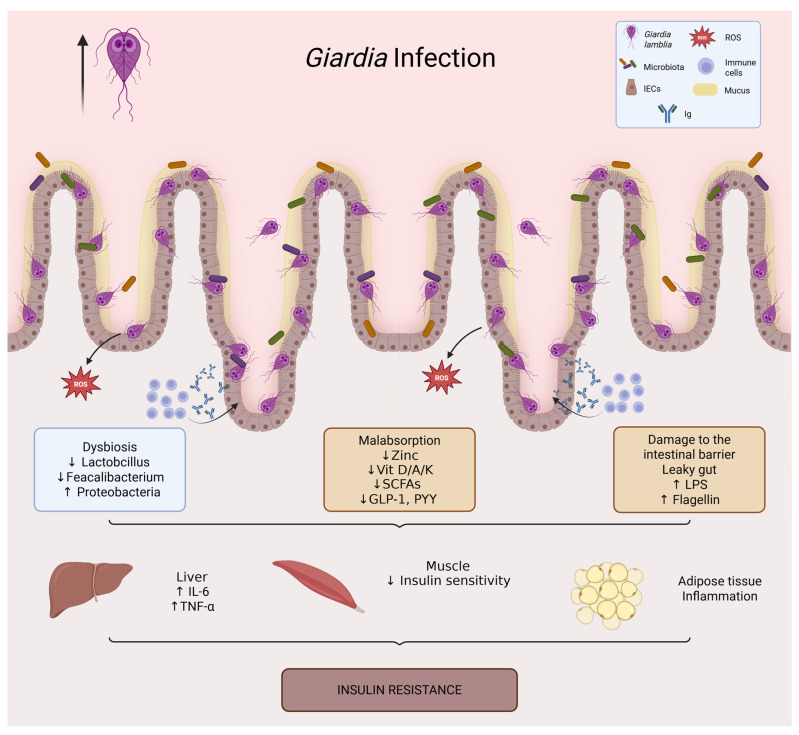
*Giardia* infection and its impact on intestinal and metabolic homeostasis. *Giardia lamblia* colonizes the small intestine through adhesion of trophozoites to the epithelial surface, leading to villus flattening, mucus degradation, and epithelial dysfunction. The infection is associated with intestinal dysbiosis (loss of *Lactobacillus* and *Faecalibacterium*, increased *Proteobacteria*), barrier disruption (increased intestinal permeability and translocation of LPS and flagellin), and micronutrient malabsorption (zinc, vitamins D/A/K, and B complex). Reduced production of short-chain fatty acids further decreases incretin secretion (GLP-1, PYY). These alterations promote systemic inflammation (↑ IL-6, TNF-α), impaired insulin signaling in muscle, and adipose tissue inflammation, ultimately contributing to insulin resistance.

Infection by *Giardia lamblia* is associated with intestinal dysbiosis (Figure 4). Unlike other pathogens that elicit severe inflammation, Giardia induces a more subclinical and persistent dysbiosis that significantly affects the microbial ecosystem’s functionality [120,121].

Like other infections, a reduction in obligate anaerobes such as *Ruminococcus*, *Roseburia*, *Faecalibacterium*, and *Clostridium leptum* has been observed. These are short-chain fatty acid producing bacteria, and their loss is accompanied by an increased relative abundance of *Proteobacteria*, including pro-inflammatory species like *Escherichia coli*, *Klebsiella*, and *Enterobacter* [28,120,122,123]. Additionally, some studies have shown alterations in the Bacteroides-Prevotella ratio, associated with changes in polysaccharide fermentation and amino acid metabolism, including the loss of dietary fiber fermentation capabilities. This may reduce the production of key microbial metabolites and contribute to immunometabolic dysregulation [124,125,126].

A hallmark of giardiasis is reduced intestinal motility and altered bile content, which can facilitate small intestinal bacterial overgrowth (SIBO), further perpetuating dysbiosis, inflammation, and malabsorption [120,127].

#### 7.6.2. Damage to the Intestinal Barrier

Although Giardia does not invade the intestinal epithelium, its direct adhesion to enterocytes via its ventral disc induces a range of mechanical and functional changes that compromise intestinal barrier integrity [118,119].

Initially, it causes epithelial disorganization. By adhering to duodenal and jejunal villi, Giardia leads to villus flattening, microvillus shortening, and actin cytoskeleton disruption. This loss of absorptive surface area reduces nutrient uptake and creates a more permeable epithelium vulnerable to the passage of luminal toxins and antigens [118,128].

Furthermore, continuous Giardia adhesion disrupts tight junctions by decreasing the expression of key proteins such as claudins, occludin, and ZO-1. Giardia also secretes proteases that degrade mucin and damage the mucus layer, reducing mechanical protection against pathogens and toxins [129,130].

Together, these alterations can facilitate the dissemination of microbial products and promote low-grade inflammatory responses that may impair insulin sensitivity, particularly in chronic or recurrent infections [131,132]. This activates receptors such as TLR4 on immune cells, hepatocytes, and adipocytes, promoting the release of pro-inflammatory cytokines (IL-6, TNF-α) that interfere with insulin receptor signaling [132]. In chronic or repeated infections, this low-grade systemic inflammation may contribute to intestinal endocrine dysfunction with lasting metabolic implications [131,132].

#### 7.6.3. Reduced Nutrient Absorption

One of the most significant consequences of Giardia infection is the disruption of nutrient absorption in the small intestine, particularly in the duodenum and jejunum—key segments for the uptake of essential micronutrients [133,134,135].

This includes zinc [136], lipids such as short-chain fatty acids [28,137], and vitamins A, B, D, E, and K, especially in children. Vitamin D is particularly important, as it regulates insulin receptor expression and modulates inflammation through the vitamin D receptor (VDR) [135,138,139,140].

## 8. Integrative Synthesis: Convergent and Pathogen-Specific Pathways Linking Diarrheagenic Infections to Insulin Resistance

Across the pathogens reviewed, three mechanistic processes repeatedly converge on insulin resistance (IR): (i) disruption of gut microbial ecology with loss of protective taxa and functional shifts in microbial metabolism, (ii) epithelial injury and/or impaired barrier integrity that facilitates the systemic dissemination of luminal microbial products, and (iii) innate immune activation with low-grade inflammatory signaling capable of interfering with insulin action. Despite this convergence, the relative contribution, anatomical locus, and persistence of these processes differ across organisms, shaping distinct mechanistic “profiles” that may influence IR risk and duration.

A first differentiating dimension is intestinal niche and injury pattern. Enteropathogenic and enterohemorrhagic *E. coli* prominently alter epithelial structure through attaching/effacing lesions and toxin-mediated effects, whereas *Salmonella enterica* relies on invasion programs (type III secretion systems) that promote inflammatory remodeling and may preferentially expand in inflamed luminal conditions [56,141]. *C. difficile* represents a toxin-driven colonic injury phenotype that is frequently precipitated by antibiotic-mediated ecological collapse, with TcdA/TcdB exerting direct cytoskeletal and junctional disruption and amplifying mucosal inflammation. In contrast, norovirus and rotavirus largely manifest as villous injury and enterocyte loss in the small intestine, producing acute malabsorption and, in some contexts, lingering post-infectious microbiota and barrier perturbations [142,143]. *Giardia lamblia* is distinctive in that it is typically noninvasive, yet can produce durable functional disruption through adhesion-mediated epithelial changes, mucus layer alterations, bile/motility perturbations, and a propensity toward small intestinal bacterial overgrowth [144].

A second differentiating dimension is the balance between dysbiosis-driven versus barrier-driven routes to IR. For bacterial pathogens such as diarrheagenic *E. coli* and *Salmonella*, inflammatory selection pressures and competitive strategies can drive pronounced community restructuring (often including Proteobacteria expansion), which may then potentiate barrier vulnerability and immunometabolic consequences [145]. In *C. difficile*, dysbiosis is commonly the initiating event (often antibiotic-associated), with toxins acting as the major amplifiers of junctional injury and inflammatory signaling [142]. For viral pathogens, epithelial injury itself is a primary trigger of malabsorption and barrier dysfunction, with dysbiosis emerging as a secondary effect that may persist beyond symptom resolution in susceptible hosts [146]. For *Giardia*, subclinical but persistent ecosystem shifts and functional malabsorption can dominate the post-infectious phenotype [127]. Collectively, these comparisons suggest that diarrheagenic pathogens can converge on IR through overlapping pathways while differing in (i) the dominant proximal driver (invasion/toxins vs. villous injury vs. adhesion-mediated dysfunction), (ii) expected inflammatory intensity, and (iii) the likelihood of persistent post-infectious alterations that may extend metabolic consequences beyond the acute diarrheal episode.

Taken together, these comparisons motivate a translational perspective on therapeutic opportunities to mitigate post-infectious dysbiosis and its potential metabolic sequelae.

## 9. Therapeutic Opportunities and Clinical Implications

The conceptual framework outlined in this review suggests that therapeutic opportunities should be considered across three complementary windows: prevention of recurrent enteric injury, mitigation of dysbiosis and barrier disruption during acute disease, and post-infectious recovery of microbiota function and epithelial homeostasis. Importantly, because both diarrheagenic infections and antibiotic exposure can perturb the gut ecosystem, an explicit translational priority is to reduce avoidable microbiota disruption while supporting restoration after clinically necessary treatments.

**Upstream prevention and exposure reduction.** A first opportunity is to lower the frequency and severity of enteric infections through measures with established public health benefit, including vaccination where available (e.g., rotavirus), improved water/sanitation and hygiene practices, and outbreak control in high-risk settings (schools, hospitals, long-term care facilities). By reducing recurrent diarrheal episodes—particularly early in life and in vulnerable populations—these strategies may indirectly limit cumulative microbiota disturbance and repeated epithelial injury that could contribute to longer-term immunometabolic consequences [147,148].

**Acute-phase management and antimicrobial stewardship.** During diarrheal illness, targeted etiologic diagnosis and guideline-concordant treatment can help balance symptom control with avoidance of unnecessary microbiota disruption. In this context, antimicrobial stewardship is not only an antimicrobial-resistance imperative but also a microbiome-preserving strategy: restricting antibiotics to situations where benefit is expected, choosing narrower-spectrum agents when appropriate, and limiting duration may reduce iatrogenic dysbiosis and improve interpretability of pathogen–insulin resistance associations in clinical and research settings [147,149].

**Post-infectious recovery and microbiota-directed approaches.** A third opportunity is to support recovery of microbiota function and barrier integrity after the acute episode, especially in individuals with persistent symptoms or high baseline metabolic risk (e.g., obesity, metabolic syndrome). Dietary strategies that increase fermentable substrates (e.g., fiber-rich patterns) and selected microbiota-directed interventions (prebiotics, probiotics/synbiotics, or postbiotic approaches) may help restore beneficial taxa and microbial metabolic capacity, although pathogen- and host-specific responses are expected and the strength of evidence varies across interventions. In parallel, attention to nutrient vulnerabilities commonly affected by enteric injury (e.g., zinc and vitamin status) may be clinically relevant in selected patients, given their roles in epithelial repair, immune regulation, and insulin sensitivity. Finally, in settings where it is clinically indicated (e.g., recurrent *C. difficile* infection), microbiota restoration strategies such as fecal microbiota transplantation may also have secondary relevance to immunometabolic outcomes, although dedicated studies are needed to determine the magnitude and durability of any metabolic effects [150,151].

Overall, these considerations highlight that the therapeutic implications of the pathogen–microbiota–barrier–inflammation axis are not limited to antimicrobial selection; they also include prevention, precision management of acute infection, and structured post-infectious recovery strategies aimed at minimizing persistent dysbiosis and its potential downstream metabolic consequences. Given that antibiotic exposure frequently co-occurs with diarrheagenic infections and can independently remodel the gut microbiome and host inflammation, we next discuss antibiotics and antimicrobial resistance as key modifiers and confounders when interpreting pathogen–IR associations.

## 10. Antibiotic Exposure and Antimicrobial Resistance as Modifiers and Confounders of the Infection–IR Axis

Antimicrobial resistance (AMR) is a major threat to global public health. It is estimated that infections caused by multidrug-resistant bacteria result in approximately 1.27 million direct deaths annually, in addition to indirectly contributing to another 5 million deaths. This phenomenon has been exacerbated by the excessive and indiscriminate use of antibiotics in clinical, veterinary, and agro-industrial contexts, which has facilitated the selection and spread of resistance genes among pathogenic and commensal bacteria [152].

Beyond the direct impact on the treatment of infectious diseases, emerging data suggest a less obvious but clinically significant relationship between prolonged antibiotic use and the development of chronic metabolic disorders, particularly insulin resistance and type 2 diabetes (T2D). This link falls within the microbiota-inflammation-metabolism axis, a field of growing interest in biomedical research [153].

Human gut microbiota plays a fundamental role in regulating immune processes, energy metabolism, and preserving the integrity of the intestinal barrier. The administration of antibiotics has been demonstrated to induce substantial alterations in the structure and diversity of this microbial community, resulting in a reduction in the abundance of beneficial species and enabling the proliferation of resistant and opportunistic bacteria. A notable effect of this phenomenon is the decline in the population of dietary fiber-fermenting bacteria, including *Faecalibacterium prausnitzii*, *Roseburia* spp., and *Bifidobacterium* spp. These bacteria play a pivotal role in the production of short-chain fatty acids (SCFAs), such as butyrate, propionate, and acetate [154].

These metabolites fulfill critical functions, with butyrate serving as the primary energy source for colonocytes and exhibiting anti-inflammatory properties by impeding the NF-κB pathway. Propionate and acetate have been observed to regulate hepatic gluconeogenesis, lipid oxidation, and insulin sensitivity. Furthermore, SCFAs have been shown to stimulate the release of incretins, such as GLP-1 and PYY, which play a regulatory role in appetite and glycemic metabolism. The depletion of these specialized bacteria and their metabolites engenders a pro-inflammatory milieu, thereby compromising intestinal barrier function and promoting the phenomenon known as “leaky gut [153]”.

Increased intestinal permeability permits the translocation of microbial components, such as lipopolysaccharide (LPS), into the bloodstream. This process activates Toll-like receptors (TLR4) in immune cells and adipocytes, promoting the release of proinflammatory cytokines (TNF-α, IL-6, IL-1β) [2,5]. These cytokines interfere with insulin signaling through serine phosphorylation of IRS-1 and inhibition of the PI3K/Akt pathway. This mechanism contributes to the establishment of a state of low-grade chronic inflammation that is directly associated with insulin resistance and metabolic dysfunction in peripheral tissues, such as the liver, skeletal muscle, and adipose tissue [153].

The loss of microbial diversity, as measured by alpha diversity indices such as Shannon or Simpson, has been associated with an increased risk of obesity, glucose intolerance, and T2D in both animal and human studies. Furthermore, species such as *Akkermansia muciniphila*, which play a role in the degradation of mucin and the fortification of the epithelial barrier, have been observed to undergo a substantial decrease in their population following exposure to antibiotics [155]. The observed reduction in these cells has been associated with diminished insulin sensitivity and heightened systemic inflammation [156].

Conversely, the prolonged utilization of antimicrobials has been demonstrated to foster the proliferation of proinflammatory opportunistic bacteria, including *Escherichia coli*, *Klebsiella* spp., *Proteus* spp., *Enterococcus* spp., and *Clostridioides difficile*. These species have been found to produce endotoxins, peptidoglycans, and flagellin, which have been demonstrated to continuously activate immune receptors and perpetuate a systemic inflammatory state. Even in the absence of overt clinical infections, this dysbiosis can perpetuate subclinical immune activation, which can impact carbohydrate metabolism and exacerbate insulin resistance [157,158].

Finally, the microbial imbalance generated by antibiotics has been shown to reduce the production of beneficial metabolites and to increase the synthesis of harmful compounds, such as trimethylamine oxide (TMAO). TMAO has been linked to vascular inflammation, insulin resistance, and cardiovascular risk [155].

Collectively, these findings underscore the notion that excessive utilization of antibiotics, by instigating intestinal dysbiosis, fosters systemic inflammation and modifies pivotal mechanisms in metabolic homeostasis. This scenario underscores the necessity for strategies that promote the judicious use of antibiotics. Such strategies must encompass not only the mitigation of bacterial resistance but also the consideration of the long-term metabolic consequences for the host.

## 11. Conclusions and Future Directions

Diarrheagenic pathogens represent a plausible and underappreciated contributor to insulin resistance (IR) through convergent effects on the gut ecosystem, epithelial integrity, and low-grade inflammatory signaling. Across bacterial, viral, and protozoal infections, the reviewed evidence supports a recurring pattern in which dysbiosis, barrier disruption, and immunometabolic perturbations may extend beyond the acute diarrheal episode, particularly in susceptible hosts and in settings of recurrent infection. Importantly, the mechanisms appear to converge while still differing in pathogen-specific drivers (e.g., invasion programs, toxins, villous injury, or adhesion-mediated dysfunction) and in the likelihood of persistent post-infectious alterations.

At the same time, this review is narrative in scope and the current literature has limitations that preclude strong causal inference in many contexts. Major gaps include heterogeneity in insulin-resistance endpoints, limited longitudinal human data, incomplete adjustment for key confounders (notably antibiotic exposure, diet, adiposity and baseline metabolic risk), and variable characterization of microbiome function beyond taxonomic shifts. These constraints underscore that the infection–IR relationship should be interpreted as biologically plausible and hypothesis-generating unless supported by robust longitudinal or interventional evidence.

Future studies should prioritize: (i) prospective cohorts linking well-phenotyped enteric infections to standardized IR outcomes with careful confounder control; (ii) mechanistic work integrating microbiome function (metabolomics/SCFA and bile-acid profiles), permeability markers, and immunometabolic signaling; (iii) identification of host factors that predict persistent post-infectious dysbiosis and metabolic vulnerability; and (iv) targeted interventions testing whether microbiota- and barrier-directed recovery strategies (including dietary approaches, selected microbiota-directed therapies, and stewardship-focused antimicrobial strategies) can attenuate post-infectious metabolic sequelae. Collectively, these efforts will clarify when and for whom diarrheagenic infections meaningfully contribute to IR risk, and which translational strategies are most likely to be effective.

## Data Availability

No new data were created or analyzed in this study. Data sharing is not applicable to this article.

## Abbreviations

The following abbreviations are used in this manuscript:

31P-MRS	Phosphorus-31 magnetic resonance spectroscopy
AGPAT	Acylglycerol-3-phosphate acyltransferase
Akt (PKB)	Protein kinase B
AMPK	AMP-activated protein kinase
AMR	Antimicrobial resistance
BOLD MRI	Blood oxygen level–dependent magnetic resonance imaging
BNIP3	BCL2/adenovirus E1B 19 kDa interacting protein 3
BNIP3L (NIX)	BNIP3-like (NIX)
CD14	Cluster of differentiation 14
Cdc42	Cell division control protein 42
DAG	Diacylglycerol
DAEC	Diffusely adherent *Escherichia coli*
DGAT	Diacylglycerol acyltransferase
DMT1	Divalent metal transporter 1
DNA	Deoxyribonucleic acid
Drp1	Dynamin-related protein 1
*E. coli*	*Escherichia coli*
EAEC	Enteroaggregative *E. coli*
EAST1	Enteroaggregative heat-stable toxin 1
EHEC	Enterohemorrhagic *E. coli* (Shiga toxin–producing *E. coli*)
EIEC	Enteroinvasive *E. coli*
EPEC	Enteropathogenic *E. coli*
ER	Endoplasmic reticulum
ETEC	Enterotoxigenic *E. coli*
*F. prausnitzii*	*Faecalibacterium prausnitzii*
GLP-1	Glucagon-like peptide 1
GLUT2	Glucose transporter type 2
GLUT4	Glucose transporter type 4
GPAT	Glycerol-3-phosphate acyltransferase
GPR41	G protein–coupled receptor 41
GPR43	G protein–coupled receptor 43
H_2_O_2_	Hydrogen peroxide
hsCRP	High-sensitivity C-reactive protein
IKKβ	IκB kinase beta
IL-1β	Interleukin-1 beta
IL-5	Interleukin-5
IL-6	Interleukin-6
IL-8	Interleukin-8
iNOS	Inducible nitric oxide synthase
IR	Insulin resistance
IRAK	Interleukin-1 receptor–associated kinase
IRS	Insulin receptor substrate
IRS1	Insulin receptor substrate 1
JNK	c-Jun N-terminal kinase
LBP	Lipopolysaccharide-binding protein
LPA	Lysophosphatidic acid
LPS	Lipopolysaccharide
LT	Heat-labile enterotoxin
ST	Heat-stable enterotoxin
MCP1 (CCL2)	Monocyte chemoattractant protein 1 (C-C motif chemokine ligand 2)
mTOR	Mechanistic target of rapamycin
mTORC2	mTOR complex 2
MyD88	Myeloid differentiation primary response 88
NAP1/BI (027)	Hypervirulent *C. difficile* strain designation (ribotype 027/NAP1/BI)
NF-κB	Nuclear factor kappa B
NLR	NOD-like receptor
NLRP3	NLR family pyrin domain containing 3
NOD2	Nucleotide-binding oligomerization domain-containing protein 2
NSP4	Non-structural protein 4 (rotavirus enterotoxin)
O_2_^•−^	Superoxide anion
PA	Phosphatidic acid
PI3K	Phosphoinositide 3-kinase
PKC	Protein kinase C
PPAR-γ	Peroxisome proliferator-activated receptor gamma
PRRs	Pattern recognition receptors
PYY	Peptide YY
RNS	Reactive nitrogen species
ROS	Reactive oxygen species
SCFAs	Short-chain fatty acids
SGLT1	Sodium–glucose cotransporter 1
SIBO	Small intestinal bacterial overgrowth
SOD	Superoxide dismutase
SPI-1	*Salmonella* pathogenicity island 1
SPI-2	*Salmonella* pathogenicity island 2
STEC	Shiga toxin–producing *Escherichia coli*
TAG	Triacylglycerol
T2D	Type 2 diabetes
TLR4	Toll-like receptor 4
TLR5	Toll-like receptor 5
TNF-α	Tumor necrosis factor alpha
TRAF6	TNF receptor–associated factor 6
TTSS (T3SS)	Type III secretion system
UPR	Unfolded protein response
VDR	Vitamin D receptor
ZIP4 (SLC39A4)	Zinc transporter ZIP4
ZnT8 (SLC30A8)	Zinc transporter 8
ZO-1	Zonula occludens 1
